# Immunotherapeutic targeting of HSP90 client proteins in BRAF-inhibitor resistant melanoma

**DOI:** 10.1186/2051-1426-3-S2-P432

**Published:** 2015-11-04

**Authors:** Ronald J Fecek, Simeng Wang, Walter J Storkus

**Affiliations:** 1 University of Pittsburgh, Pittsburgh, PA, USA

## 

Therapy options for patients with advanced stage melanoma have improved steadily over the past decade, with recent clinical successes noted for BRAF inhibitors (BRAFi). BRAFi such as dabrafenib are highly specific for BRAF mutant, BRAF^V600E^, which is expressed in approximately 50% of melanomas. Although treatment with BRAFi is highly efficacious with substantial tumor regression and increased patient survival, the response is short-lived, with treatment-refractory progressive disease developing as early as six months. Refractory progressive disease develops in association with tumor cell adoption of alternate signaling pathways (linked to tumor cell (over)expressed BRAFi-resistance associated molecules (BRAFi-RAM) such as FGFR3, MEK, PDGFRb, SRC, and STAT3, among others) supporting their continued survival, growth, and metastatic potential. It is worthwhile noting that each of these molecules represents a “client” protein of HSP90, a molecular chaperone commonly overexpressed in human melanomas where it serves to post-translationally stabilize a broad range of clients. Treatment of melanoma cells *in vitro* or *in vivo* with HSP90 inhibitor (HSP90i), leads to the rapid proteasome-dependent degradation of HSP90 client proteins, with the resulting peptides used to load MHC class I complexes on the tumor cell surface, thereby conditionally-enhancing specific CD8+ T cell recognition. In this study, we hypothesize that specific vaccination against BRAFi-RAM in combination with systemic HSP90i treatment will promote tumor regression and increase overall survival in a BRAFi-resistant melanoma mouse model. By treating with increasing concentrations of dabrafenib, we have selected for and maintained a dabrafenib-resistant BP (*BRAF^V600E^; PTEN-/-*) mouse melanoma cell line (BPR20) that demonstrates upregulation of BRAFi-RAM and increased activation of receptor tyrosine kinase signaling pathways. Upregulated BRAFi-RAM in BPR20 underwent proteasome-dependent degradation when treated with ganetespib, an HSP90i. Furthermore, a proportion of the upregulated BRAFi-RAM were immunogenic. Specific CD8+ T cells were generated when wild-type C57Bl/6 mice were vaccinated with IL-12-expressing dendritic cells pulsed with a BRAFi-RAM peptide pool, and those CD8+ T cells demonstrated enhanced specific reactivity to syngeneic cells presenting relevant antigen as revealed by IFNg release assays. These results suggest that a polyepitope vaccine based on BRAFi-resistance associated HSP90 client proteins could define a novel immunotherapeutic strategy for the (co)treatment of patients with advanced-stage melanomas.

